# Supplementary material for: Stress-induced vagal activity influences anxiety-relevant prefrontal and amygdala neuronal oscillations in male mice
Source: Nat Commun. 2024 Jan 9;15:183. doi: 10.1038/s41467-023-44205-y (PMC10776769; doi:10.1038/s41467-023-44205-y)
Supplement: Supplementary file 1 — Supplementary Figures [file 41467_2023_44205_MOESM1_ESM.pdf]

Supplementary Figures for

Stress-induced vagal activity influences anxiety-relevant prefrontal and amygdala neuronal oscillations in male mice

Toya Okonogi, Nahoko Kuga, Musashi Yamakawa, Tasuku Kayama, Yuji Ikegaya, Takuya Sasaki

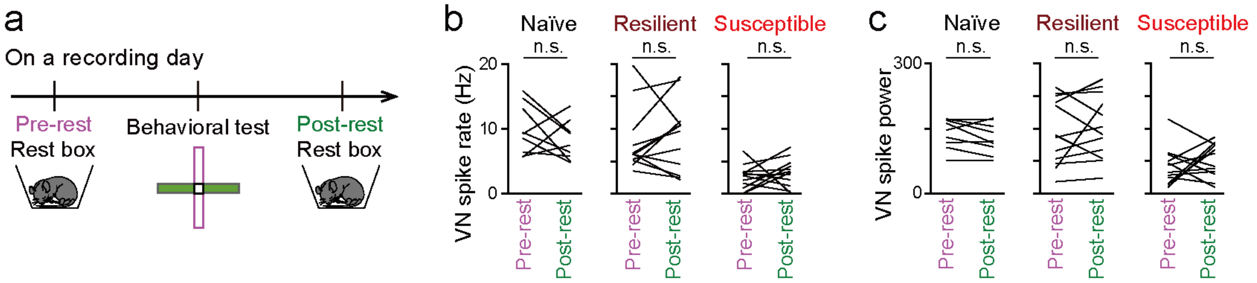

Supplementary Fig. 1: No significant differences in VN spike activity during quiescent periods in the rest periods between before and after the EPM test.

a Mice first rested in their home cage for at least 30 min (pre-rest), performed an EPM test for 10–20 min, and again rested in the same home cage (post-rest) for at least 30 min. **b** Comparisons of VN spike rates during quiescent periods between the pre-rest and post-rest periods (n = 9, 12, and 12 mice). Each line represents each mouse. naïve: $t_8 = 1.08$, $P = 0.31$; resilient: $t_{11} = 0.66$, $P = 0.52$; susceptible: $t_{11} = 0.77$, $P = 0.46$, two-sided paired t-test. **c** Comparisons of VN spike power during quiescent periods between the pre-rest and post-rest periods (n = 9, 12, and 12 mice). Each line represents each mouse. naïve: $t_8 = 1.64$, $P = 0.14$; resilient: $t_{11} = 0.66$, $P = 0.52$; susceptible: $t_{11} = 0.84$, $P = 0.42$, two-sided paired t-test. Source data are provided as a Source Data file.

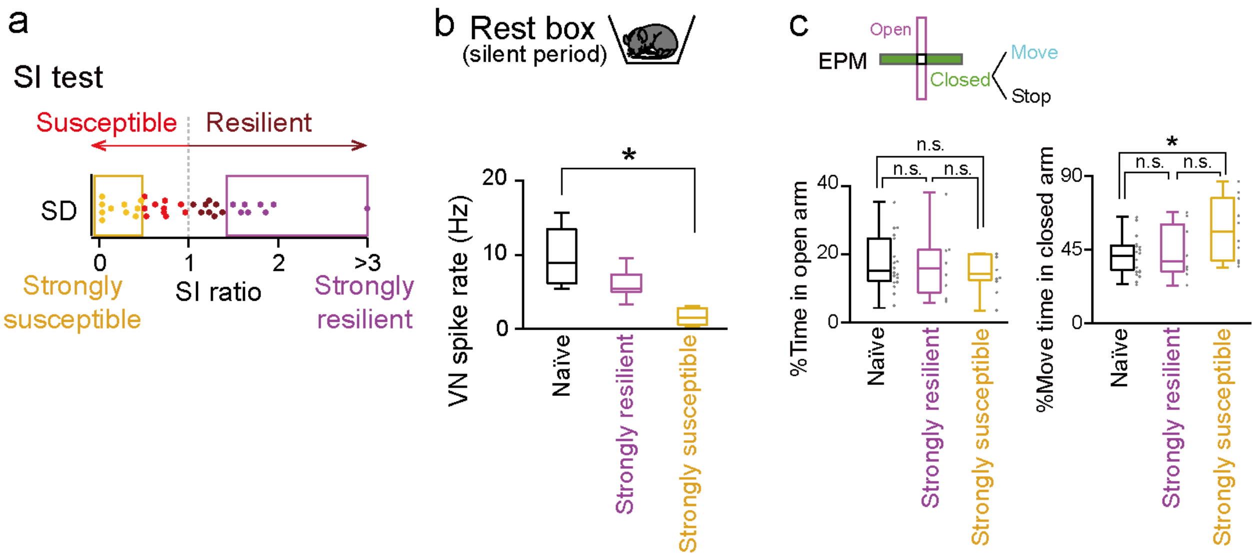

Supplementary Fig. 2: The same results of VN spike patterns were obtained from strongly stress-resilient and stress-susceptible mice.

a Stress-resilient and stress-susceptible mice were evenly divided into two subgroups based on their SI ratios. Strongly resilient mice (*n* = 8 mice) and strongly susceptible mice (*n* = 10 mice) were defined. **b** VN spike rates during quiescent periods in a rest box (*n* = 9, 6, and 5 mice). Box plots show center line as median, box limits as the 25th percentile and the 75th percentile, whiskers as minimum to maximum values that are not outliers. $F_{2,19}$ = 12.38, *P* = 0.00050, one-way ANOVA; naïve vs strongly resilient, *P* = 0.077, naïve vs strongly susceptible, **P* = 0.00030, strongly resilient vs strongly susceptible, *P* = 0.0545, two-sided Tukey's test. **c** (Left) The percentages of time spent in the open arms to the total recording time (*n* = 18, 8, and 10 mice). Box plots show center line as median, box limits as the 25th percentile and the 75th percentile, whiskers as minimum to maximum values. (Right) The percentage of move states in the closed arms (*n* = 18, 8, and 10 mice). Box plots show center line as median, box limits as the 25th percentile and the 75th percentile, whiskers as minimum to maximum values that are not outliers. Naïve vs strongly susceptible, **P* = 0.028, two-sided Tukey's test. Source data are provided as a Source Data file.

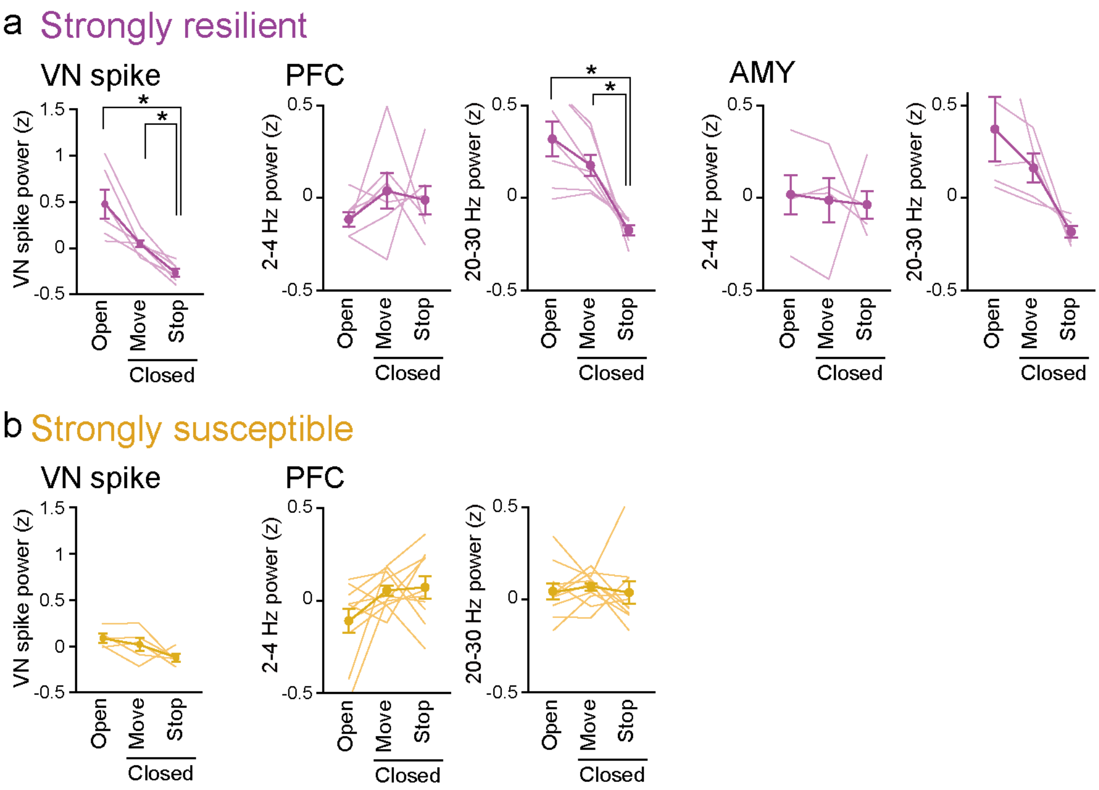

Supplementary Fig. 3: The same results of PFC LFP patterns were obtained from strongly stress-resilient and stress-susceptible mice.

a (Leftmost) VN spike power in the open arms and closed arms in strongly resilient mice (n = 6 mice). Each thin line represents each mouse. Data are presented as mean ± SEM. open vs closed move, t_5 = 3.33, P = 0.060; open vs closed stop, t_5 = 5.83, *P = 0.0063; closed move vs closed stop, t_5 = 6.00, *P = 0.0055, two-sided paired t-test followed by Bonferroni correction. (Right) Comparisons of PFC and AMY 2–4 Hz and 20–30 Hz power in strongly resilient mice (n = 7 and 5 mice). Each thin line represents each mouse. Data are presented as mean ± SEM. PFC: 2–4 Hz: open vs closed move, t_6 = 1.57, P = 0.50; open vs closed stop, t_6 = 1.12, P = 0.92; closed move vs closed stop, t_6 = 0.31, P > 0.99; 20–30 Hz: open vs closed move, t_6 = 2.95, P = 0.078; open vs closed stop, t_6 = 4.40, *P = 0.014; closed move vs closed stop, t_6 = 4.45, *P = 0.013; AMY: 2–4 Hz: open vs closed move, t_4 = 0.95, P > 0.99; open vs closed stop, t_4 = 0.31, P > 0.99; closed move vs closed stop, t_4 = 0.13, P > 0.99; 20–30 Hz: open vs closed move, t_4 = 1.46, P = 0.66; open vs closed stop, t_4 = 2.64, P = 0.17; closed move vs closed stop, t_4 = 3.28, P = 0.091, two-sided paired t-test followed by Bonferroni correction. **b** (Left) Similar to a but for strongly susceptible mice (n = 5 mice). open vs closed move, t_4 = 1.22, P = 0.87; open vs closed stop, t_4 = 3.25, P = 0.095; closed move vs closed stop, t_4 = 1.28, P = 0.81, two-sided paired t-test followed by Bonferroni correction. (Right) Similar to a but for strongly susceptible mice (n = 10 mice). 2–4 Hz: open vs closed move, t_9 = 2.10, P = 0.20; open vs closed stop, t_9 = 1.51, P = 0.49; closed move vs closed stop, t_9 = 0.30, P > 0.99; 20–30 Hz: open vs closed move, t_9 = 0.40, P > 0.99; open vs closed stop, t_9 = 0.13, P > 0.99; closed move vs closed stop, t_9 = 0.38, P > 0.99, two-sided paired t-test followed by Bonferroni correction. Source data are provided as a Source Data file.

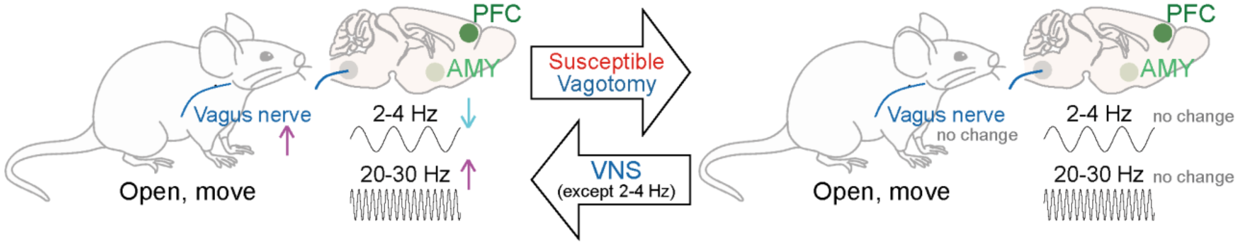

Supplementary Fig. 4: A schematic illustration summarizing this study.

(Left) In an open environment or during movement, naïve mice or stress-resilient mice exhibit increased VN spikes, decreased 2–4 Hz power, and increased 20–30 Hz power in the PFC. The AMY also shows increased 20–30 Hz power. (Right) Stress-susceptible mice or mice with vagotomy did not exhibit such physiological changes. Chronic VNS restores VN spikes and 20–30 Hz power in the PFC and AMY (from right to left).
