# Supplementary material for: Stress-induced vagal activity influences anxiety-relevant prefrontal and amygdala neuronal oscillations in male mice
Source: Nat Commun. 2024 Jan 9;15:183. doi: 10.1038/s41467-023-44205-y (PMC10776769; doi:10.1038/s41467-023-44205-y)
Supplement: Supplementary file 2 — Reporting Summary [file 41467_2023_44205_MOESM2_ESM.pdf]

Corresponding author(s): Takuya Sasaki

Last updated by author(s): Nov 22, 2023

Reporting Summary

Nature Portfolio wishes to improve the reproducibility of the work that we publish. This form provides structure for consistency and transparency in reporting. For further information on Nature Portfolio policies, see our Editorial Policies and the Editorial Policy Checklist.

Statistics

For all statistical analyses, confirm that the following items are present in the figure legend, table legend, main text, or Methods section.

n/a	Confirmed	
☐	☒	The exact sample size (n) for each experimental group/condition, given as a discrete number and unit of measurement
☒	☐	A statement on whether measurements were taken from distinct samples or whether the same sample was measured repeatedly
☐	☒	The statistical test(s) used AND whether they are one- or two-sided<br>*Only common tests should be described solely by name; describe more complex techniques in the Methods section.*
☒	☐	A description of all covariates tested
☐	☒	A description of any assumptions or corrections, such as tests of normality and adjustment for multiple comparisons
☐	☒	A full description of the statistical parameters including central tendency (e.g. means) or other basic estimates (e.g. regression coefficient) AND variation (e.g. standard deviation) or associated estimates of uncertainty (e.g. confidence intervals)
☐	☒	For null hypothesis testing, the test statistic (e.g. F, t, r) with confidence intervals, effect sizes, degrees of freedom and P value noted<br>*Give P values as exact values whenever suitable.*
☒	☐	For Bayesian analysis, information on the choice of priors and Markov chain Monte Carlo settings
☒	☐	For hierarchical and complex designs, identification of the appropriate level for tests and full reporting of outcomes
☒	☐	Estimates of effect sizes (e.g. Cohen's d, Pearson's r), indicating how they were calculated

Our web collection on statistics for biologists contains articles on many of the points above.

Software and code

Policy information about availability of computer code

Data collection	A Cereplex Direct data acquisition system (Blackrock Microsystems) was used for electrophysiological data collection. CMOS camera (MCM4350, Gazo) attached on the ceiling was used for recording animal's behavior.
Data analysis	For All electrophysiological data analysis, Matlab R2022a was used. For spike sorting, the graphical cluster-cutting software MClust4.3.02 was used. Animal's trajectories in images were manually extracted by Image J1.45.

For manuscripts utilizing custom algorithms or software that are central to the research but not yet described in published literature, software must be made available to editors and reviewers. We strongly encourage code deposition in a community repository (e.g. GitHub). See the Nature Portfolio guidelines for submitting code & software for further information.

Data

Policy information about availability of data

All manuscripts must include a data availability statement. This statement should provide the following information, where applicable:

- Accession codes, unique identifiers, or web links for publicly available datasets
- A description of any restrictions on data availability
- For clinical datasets or third party data, please ensure that the statement adheres to our policy

All data needed to evaluate the conclusions are described in the manuscript and/or Supplementary Materials. Original physiological datasets are provided on Mendeley Data (https://data.mendeley.com/datasets/6yy34xkfmb/1). Source data are provided with this paper.

Research involving human participants, their data, or biological material

Policy information about studies with human participants or human data. See also policy information about sex, gender (identity/presentation), and sexual orientation and race, ethnicity and racism.

Reporting on sex and gender	N/A
Reporting on race, ethnicity, or other socially relevant groupings	N/A
Population characteristics	N/A
Recruitment	N/A
Ethics oversight	N/A

Note that full information on the approval of the study protocol must also be provided in the manuscript.

Field-specific reporting

Please select the one below that is the best fit for your research. If you are not sure, read the appropriate sections before making your selection.

☒ Life sciences ☐ Behavioural & social sciences ☐ Ecological, evolutionary & environmental sciences

For a reference copy of the document with all sections, see nature.com/documents/nr-reporting-summary-flat.pdf

Life sciences study design

All studies must disclose on these points even when the disclosure is negative.

Sample size	Sample sizes were determined from prospective power calculations based on data from comparable experiments.
Data exclusions	For behavioral tests, all mice were used for analyses. For electrophysiological tests, no exlcusions of mice, except those that lost recording connections, were performed.
Replication	Independent behavioral and electrophysiological experiments other than those in the reporting summary were repeated four times to get the similar results from independent mouse groups including four mice. All attempts at replication were successful.
Randomization	For all experiments, mice were randomly assigned to experimental groups by sampling across different litters.
Blinding	All the data and influences in this study were analyzed and generated completely independently. Hence blinding was not applicable as there was no bias present.

Reporting for specific materials, systems and methods

We require information from authors about some types of materials, experimental systems and methods used in many studies. Here, indicate whether each material, system or method listed is relevant to your study. If you are not sure if a list item applies to your research, read the appropriate section before selecting a response.

Materials & experimental systems

n/a	Involved in the study	
☐	☒	Antibodies
☒	☐	Eukaryotic cell lines
☒	☐	Palaeontology and archaeology
☐	☒	Animals and other organisms
☒	☐	Clinical data
☒	☐	Dual use research of concern
☒	☐	Plants

Methods

n/a	Involved in the study	
☒	☐	ChIP-seq
☒	☐	Flow cytometry
☒	☐	MRI-based neuroimaging

Antibodies

Antibodies used	Primary antibodies: mouse anti-c-Fos antibody (EnCor Biotechnology, Cat# MCA-2H2, 1:1000)

Antibodies used	Fluorophore-conjugated secondary antibodies: anti-mouse IgG antibody Alexa 488 (Invitrogen, Cat# A-11029, 1:500)
Validation	primary mouse anti-c-Fos antibody (https://encorbio.com/product/mca-2h2/)<br>secondary mouse IgG antibody (https://www.thermofisher.com/antibody/product/Goat-anti-Mouse-IgG-H-L-Highly-Cross-Adsorbed-Secondary-Antibody-Polyclonal/A-11029)

Animals and other research organisms

Policy information about studies involving animals; ARRIVE guidelines recommended for reporting animal research, and Sex and Gender in Research

Laboratory animals	Male C57BL/6J mice (8–10 weeks old) with preoperative weights of 29–35 g were used for all data sampling. In addition, male CD-1 mice (more than 13 weeks old) with weights of 40–50 g were used as aggressor mice that imposed social defeat stress. They were individually housed and maintained on a 12-h light/12-h dark schedule under housing conditions at 23 ± 1°C with relative humidity of 50 ± 5% with lights off at 8:00 AM. All mice were purchased from a vendor.
Wild animals	No wild animals were used.
Reporting on sex	Only male mice were used in this study because the experimental paradigms of social defeat stress employed in this study have been established for male mice.
Field-collected samples	No field-collceted samples were used.
Ethics oversight	All experiments were performed with the approval of the animal experimental ethics committee at the University of Tokyo (approval number: P29-14) and the committee on animal experiments at Tohoku University (approval number: 2022 PhA-004) and in accordance with the NIH guidelines for the care and use of animals.

Note that full information on the approval of the study protocol must also be provided in the manuscript.

Plants

Seed stocks	N/A
Novel plant genotypes	N/A
Authentication	N/A
